# 2,3-Diamino­phenazine tetra­hydrate

**DOI:** 10.1107/S1600536808009598

**Published:** 2008-05-07

**Authors:** Xiao-Feng Li, Yan An, Yan-Sheng Yin

**Affiliations:** a Institute of Marine Materials and Engineering, Shanghai Maritime University, Shanghai 200135, People’s Republic of China

## Abstract

The title compound, C_12_H_10_N_4_·4H_2_O, was obtained from a room-temperature solution of *o*-phenyl­enediamine and copper acetate. In the crystal structure, there are significant π–π stacking inter­actions, with a centroid–centroid separation of 3.575 (2) Å. In addition, inter­molecular O—H⋯O, N—H⋯O, N—H⋯N and O—H⋯N hydrogen bonds link 2,3-diamino­phenazine mol­ecules and water mol­ecules, forming a three-dimensional framework.

## Related literature

For related literature, see: Brownstein & Enright (1995 ▶); Doyle *et al.* (2001 ▶); Chłopek *et al.* (2005 ▶).
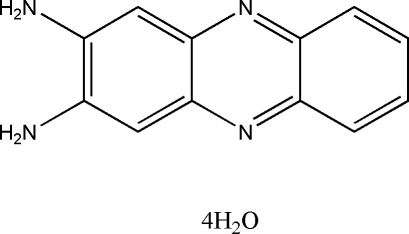


## Experimental

### 

#### Crystal data

C_12_H_10_N_4_·4H_2_O
*M* *_r_* = 282.30
Orthorhombic, 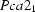

*a* = 16.7593 (18) Å
*b* = 18.1200 (19) Å
*c* = 4.7834 (5) Å
*V* = 1452.6 (3) Å^3^
*Z* = 4
Mo *K*α radiation
μ = 0.10 mm^−1^
*T* = 293 (2) K
0.37 × 0.32 × 0.23 mm

#### Data collection

Bruker SMART APEX area-detector diffractometer
Absorption correction: multi-scan (*SADABS*; Sheldrick, 1996 ▶) *T*_min_ = 0.965, *T*_max_ = 0.977
7735 measured reflections
1608 independent reflections
1432 reflections with *I* > 2σ(*I*)
*R*_int_ = 0.022

#### Refinement

*R*[*F* ^2^ > 2σ(*F* ^2^)] = 0.048
*wR*(*F* ^2^) = 0.140
*S* = 1.14
1608 reflections
225 parameters
17 restraints
H atoms treated by a mixture of independent and constrained refinement
Δρ_max_ = 0.29 e Å^−3^
Δρ_min_ = −0.12 e Å^−3^

### 

Data collection: *SMART* (Bruker, 2001 ▶); cell refinement: *SAINT* (Bruker, 2001 ▶); data reduction: *SAINT*; program(s) used to solve structure: *SHELXS97* (Sheldrick, 2008 ▶); program(s) used to refine structure: *SHELXL97* (Sheldrick, 2008 ▶); molecular graphics: *SHELXTL* (Sheldrick, 2008 ▶); software used to prepare material for publication: *SHELXTL*.

## Supplementary Material

Crystal structure: contains datablocks I, global. DOI: 10.1107/S1600536808009598/lh2601sup1.cif

Structure factors: contains datablocks I. DOI: 10.1107/S1600536808009598/lh2601Isup2.hkl

Additional supplementary materials:  crystallographic information; 3D view; checkCIF report

## Figures and Tables

**Table 1:** Hydrogen-bond geometry (Å, °)

*D*—H⋯*A*	*D*—H	H⋯*A*	*D*⋯*A*	*D*—H⋯*A*
N4—H4*B*⋯O2*W*^i^	0.895 (10)	2.218 (12)	3.105 (5)	171 (3)
N4—H4*C*⋯N4^ii^	0.90 (3)	2.58 (3)	3.198 (3)	126 (3)
N3—H3*B*⋯O1*W*^iii^	0.906 (10)	2.165 (16)	3.048 (6)	165 (4)
N3—H3*C*⋯N3^ii^	0.895 (11)	2.33 (2)	3.122 (4)	147 (3)
O4*W*—H4*WA*⋯O4*W*^iv^	0.855 (19)	2.017 (19)	2.871 (3)	176 (4)
O4*W*—H4*WB*⋯N2	0.867 (17)	1.924 (19)	2.787 (3)	173 (4)
O3*W*—H3*WB*⋯N1	0.872 (19)	1.96 (3)	2.801 (3)	161 (6)
O2*W*—H2*WA*⋯O4*W*^v^	0.84 (2)	2.01 (2)	2.843 (4)	178 (5)
O1*W*—H1*WA*⋯O1*W*^vi^	0.84 (6)	2.15 (6)	2.860 (7)	142 (6)
O2*W*—H2*WB*⋯O2*W*^vii^	0.87 (4)	1.97 (4)	2.812 (5)	161 (3)
O1*W*—H1*WB*⋯O3*W*	0.85 (3)	2.09 (3)	2.882 (6)	155 (5)

Symmetry codes: (i) 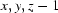; (ii) 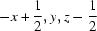; (iii) 
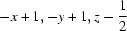
; (iv) 
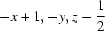
; (v) 
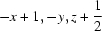
; (vi) 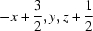; (vii) 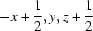.

**Fig. 1.**
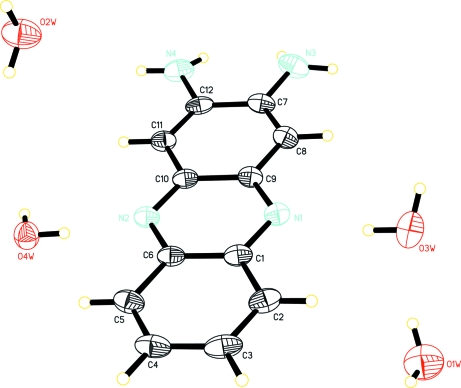
The asymmetric unit of (I).

**Fig. 2.**
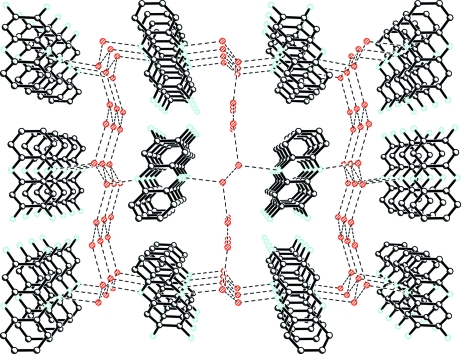
Part of the crystal structure viewed along the c-axis. Dashed lines are drawn between the donor and acceptor atoms of the hydrogen bonds but H atoms are not showm.

## supplementary crystallographic information

### Comment

The crystal structures of phenazinediamine (Doyle, *et al.*, 2001) and examples of its derivatives (Brownstein, *et al.*, 1995; Krzysztof, *et al.*, 2005) have been published. As part of our studies of these types of compounds we report here the crystal structure of the title compound (I) which was synthesized at room temperature using *o*-Phenylenediamine and copper acetate.

In compound (I), the asymmetric unit contains a 2,3-Diamino-phenazine molecule and four water molecules (Fig. 1). In the crystal structure, 2,3-Diamino-phenazine molecules related by unit cell translations along the *c* axis form moderately strong π···π stacking interactions (*Cg*1···.*Cg*2(*x*, *y*, -1 + *z*) and *Cg*1···*Cg*3(*x*, *y*, 1 + *z*) = 3.575 (2) Å, where *Cg*1, *Cg*2 and *Cg*3 are the centroids defined by ring atoms N1/N2/C1/C6/C9/C10, C1—C6 and C7—C12, respectively). In addition, water molecules and 2,3-Diamino-phenazine molecules are linked by O—H···N, O—H···O, N—H···N and H—H···O hydrogen bonds to form a three-dimensional network (Table 1 & Fig.2).

### Experimental

A mixture of *o*-Phenylenediamine(0.5 mmol, 0.054 g), Cu(CH_3_COO)_2_ (0.5 mmol,0.099 g), NaOH (1 mmol, 0.04 g), and water (10 ml) was placed in a 20 ml vial, stirring in air for 1 h. It was then sealed for 1 week and the resulting black block-shaped single crystals were collected. Yield: 67%. C&H analysis for C_12_H_18_N_4_O_4_ (found/calc): C, 51.03(51.06), H, 6.39(6.43).

### Refinement

In the absence of significant anomalous dispersion effects the Friedel pairs were merged. The H atoms were placed in calculated positions in the riding-model approximation (C—H 0.93 Å, N—H 0.90 Å), with their temperature factors were set to 1.2 times those of the equivalent isotropic temperature factors of the parent atoms. The water H atoms were located in difference Fourier maps and refined isotropically with distance restrains of O—H = 0.85 (2) and H···H = 1.39 (1) Å.

### Crystal data

**Table:**

C_12_H_10_N_4_·4H_2_O	*F*_000_ = 600
*M**_r_* = 282.30	*D*_x_ = 1.291 Mg m^−^^3^
Orthorhombic, *P**c**a*2_1_	Mo *K*α radiation λ = 0.71073 Å
Hall symbol: P 2c -2ac	Cell parameters from 3569 reflections
*a* = 16.7593 (18) Å	θ = 2.7–24.3º
*b* = 18.1200 (19) Å	µ = 0.10 mm^−^^1^
*c* = 4.7834 (5) Å	*T* = 293 (2) K
*V* = 1452.6 (3) Å^3^	Block, black
*Z* = 4	0.37 × 0.32 × 0.23 mm

### Data collection

**Table:**

Bruker SMART APEX area-detector diffractometer	1608 independent reflections
Radiation source: fine-focus sealed tube	1432 reflections with *I* > 2σ(*I*)
Monochromator: graphite	*R*_int_ = 0.022
*T* = 293(2) K	θ_max_ = 26.0º
φ and ω scans	θ_min_ = 1.1º
Absorption correction: multi-scan(SADABS; Sheldrick, 1996)	*h* = −20→18
*T*_min_ = 0.965, *T*_max_ = 0.977	*k* = −19→22
7735 measured reflections	*l* = −5→5

### Refinement

**Table:**

Refinement on *F*^2^	Secondary atom site location: difference Fourier map
Least-squares matrix: full	Hydrogen site location: inferred from neighbouring sites
*R*[*F*^2^ > 2σ(*F*^2^)] = 0.048	H atoms treated by a mixture of independent and constrained refinement
*wR*(*F*^2^) = 0.140	*w* = 1/[σ^2^(*F*_o_^2^) + (0.0963*P*)^2^] where *P* = (*F*_o_^2^ + 2*F*_c_^2^)/3
*S* = 1.14	(Δ/σ)_max_ < 0.001
1608 reflections	Δρ_max_ = 0.29 e Å^−^^3^
225 parameters	Δρ_min_ = −0.12 e Å^−^^3^
17 restraints	Extinction correction: none
Primary atom site location: structure-invariant direct methods	

### Special details

**Table:**

Geometry. All e.s.d.'s (except the e.s.d. in the dihedral angle between two l.s. planes) are estimated using the full covariance matrix. The cell e.s.d.'s are taken into account individually in the estimation of e.s.d.'s in distances, angles and torsion angles; correlations between e.s.d.'s in cell parameters are only used when they are defined by crystal symmetry. An approximate (isotropic) treatment of cell e.s.d.'s is used for estimating e.s.d.'s involving l.s. planes.
Refinement. Refinement of *F*^2^ against ALL reflections. The weighted *R*-factor *wR* and goodness of fit *S* are based on *F*^2^, conventional *R*-factors *R* are based on *F*, with *F* set to zero for negative *F*^2^. The threshold expression of *F*^2^ > σ(*F*^2^) is used only for calculating *R*-factors(gt) *etc*. and is not relevant to the choice of reflections for refinement. *R*-factors based on *F*^2^ are statistically about twice as large as those based on *F*, and *R*- factors based on ALL data will be even larger.

### Fractional atomic coordinates and isotropic or equivalent isotropic displacement parameters (Å^2^)

**Table:**

	*x*	*y*	*z*	*U*_iso_*/*U*_eq_	
N1	0.51508 (13)	0.31719 (12)	0.3965 (5)	0.0417 (6)	
N2	0.51933 (12)	0.16406 (12)	0.2821 (6)	0.0410 (5)	
C10	0.46946 (14)	0.20894 (15)	0.1460 (6)	0.0393 (6)	
C11	0.41763 (15)	0.18021 (16)	−0.0623 (7)	0.0443 (7)	
H11A	0.4184	0.1299	−0.1008	0.053*	
C1	0.56542 (15)	0.27142 (16)	0.5338 (6)	0.0418 (7)	
C12	0.36660 (15)	0.22488 (17)	−0.2076 (6)	0.0445 (7)	
C8	0.41408 (16)	0.33188 (16)	0.0507 (7)	0.0456 (7)	
H8A	0.4123	0.3823	0.0873	0.055*	
C9	0.46739 (15)	0.28738 (15)	0.2042 (6)	0.0394 (6)	
N4	0.31334 (15)	0.19700 (17)	−0.3960 (7)	0.0603 (8)	
H4B	0.3134 (18)	0.1476 (6)	−0.403 (10)	0.072*	
H4C	0.2916 (19)	0.2287 (16)	−0.520 (7)	0.072*	
N3	0.30987 (17)	0.34664 (19)	−0.2858 (6)	0.0630 (8)	
H3B	0.315 (2)	0.3961 (7)	−0.263 (12)	0.076*	
H3C	0.2912 (19)	0.333 (2)	−0.454 (4)	0.076*	
C5	0.62032 (15)	0.14891 (17)	0.6301 (7)	0.0498 (7)	
H5A	0.6222	0.0985	0.5942	0.060*	
C6	0.56701 (14)	0.19474 (15)	0.4760 (6)	0.0401 (6)	
C2	0.61674 (16)	0.30014 (19)	0.7428 (7)	0.0512 (8)	
H2A	0.6163	0.3504	0.7828	0.061*	
C7	0.36499 (15)	0.30342 (16)	−0.1501 (6)	0.0446 (7)	
C4	0.66820 (16)	0.1785 (2)	0.8286 (7)	0.0561 (8)	
H4A	0.7025	0.1480	0.9289	0.067*	
C3	0.66680 (18)	0.25428 (18)	0.8851 (7)	0.0576 (9)	
H3A	0.7004	0.2736	1.0213	0.069*	
O4W	0.54392 (14)	0.01642 (12)	0.1437 (6)	0.0592 (6)	
O3W	0.53446 (19)	0.47020 (15)	0.4439 (8)	0.0798 (8)	
O2W	0.29410 (18)	0.02670 (19)	0.5763 (7)	0.0818 (8)	
O1W	0.7032 (3)	0.4897 (3)	0.3542 (10)	0.1042 (11)	
H4WB	0.541 (2)	0.0631 (11)	0.182 (8)	0.088 (14)*	
H3WB	0.535 (3)	0.4238 (14)	0.396 (16)	0.16 (3)*	
H4WA	0.516 (2)	0.0085 (18)	−0.004 (7)	0.070 (12)*	
H3WA	0.4832 (14)	0.481 (2)	0.455 (16)	0.14 (2)*	
H2WA	0.3416 (11)	0.014 (2)	0.601 (11)	0.102 (16)*	
H1WA	0.723 (3)	0.469 (4)	0.495 (12)	0.22 (4)*	
H2WB	0.266 (2)	0.016 (2)	0.724 (8)	0.093 (16)*	
H1WB	0.6547 (14)	0.478 (2)	0.330 (13)	0.11 (2)*	

### Atomic displacement parameters (Å^2^)

**Table:**

	*U*^11^	*U*^22^	*U*^33^	*U*^12^	*U*^13^	*U*^23^
N1	0.0396 (11)	0.0510 (12)	0.0344 (13)	−0.0021 (9)	0.0026 (10)	−0.0015 (11)
N2	0.0359 (11)	0.0526 (12)	0.0346 (12)	0.0009 (9)	0.0026 (11)	−0.0002 (11)
C10	0.0319 (12)	0.0550 (14)	0.0311 (14)	−0.0024 (10)	0.0056 (12)	0.0001 (12)
C11	0.0373 (13)	0.0571 (15)	0.0386 (15)	−0.0018 (12)	−0.0006 (13)	−0.0059 (13)
C1	0.0337 (13)	0.0606 (16)	0.0309 (15)	−0.0032 (11)	0.0033 (11)	0.0012 (12)
C12	0.0302 (13)	0.0741 (18)	0.0293 (14)	−0.0081 (12)	0.0057 (11)	−0.0011 (14)
C8	0.0444 (14)	0.0551 (14)	0.0375 (16)	0.0061 (12)	0.0035 (13)	0.0010 (13)
C9	0.0361 (12)	0.0509 (14)	0.0313 (16)	0.0018 (11)	0.0035 (11)	−0.0008 (12)
N4	0.0465 (14)	0.092 (2)	0.0428 (16)	−0.0086 (13)	−0.0108 (13)	−0.0011 (17)
N3	0.0539 (15)	0.089 (2)	0.0463 (17)	0.0191 (14)	−0.0071 (14)	0.0025 (15)
C5	0.0394 (13)	0.0697 (17)	0.0401 (17)	0.0079 (12)	0.0028 (13)	0.0071 (16)
C6	0.0303 (12)	0.0586 (15)	0.0315 (15)	−0.0004 (10)	0.0021 (12)	0.0022 (13)
C2	0.0423 (15)	0.0733 (18)	0.0379 (17)	−0.0112 (13)	0.0007 (13)	−0.0048 (16)
C7	0.0357 (13)	0.0699 (18)	0.0282 (15)	0.0063 (12)	0.0034 (12)	0.0024 (13)
C4	0.0358 (14)	0.090 (2)	0.0421 (18)	0.0057 (15)	−0.0020 (13)	0.0131 (17)
C3	0.0379 (15)	0.098 (3)	0.0366 (16)	−0.0096 (15)	−0.0058 (14)	0.0019 (17)
O4W	0.0671 (14)	0.0536 (12)	0.0569 (15)	0.0009 (10)	−0.0081 (13)	0.0014 (12)
O3W	0.103 (2)	0.0588 (14)	0.078 (2)	−0.0099 (13)	−0.003 (2)	−0.0042 (14)
O2W	0.0642 (17)	0.118 (2)	0.0636 (18)	0.0089 (16)	−0.0007 (16)	0.0027 (17)
O1W	0.097 (2)	0.123 (3)	0.092 (3)	−0.006 (2)	0.007 (2)	0.011 (2)

### Geometric parameters (Å, °)

**Table:**

N1—C9	1.333 (3)	N3—H3B	0.906 (10)
N1—C1	1.353 (3)	N3—H3C	0.895 (11)
N2—C10	1.335 (3)	C5—C4	1.353 (5)
N2—C6	1.345 (4)	C5—C6	1.425 (4)
C10—C11	1.421 (4)	C5—H5A	0.9300
C10—C9	1.449 (4)	C2—C3	1.363 (4)
C11—C12	1.367 (4)	C2—H2A	0.9300
C11—H11A	0.9300	C4—C3	1.400 (4)
C1—C6	1.417 (4)	C4—H4A	0.9300
C1—C2	1.417 (4)	C3—H3A	0.9300
C12—N4	1.366 (4)	O4W—H4WB	0.867 (17)
C12—C7	1.450 (4)	O4W—H4WA	0.855 (19)
C8—C7	1.366 (4)	O3W—H3WB	0.872 (19)
C8—C9	1.410 (4)	O3W—H3WA	0.883 (19)
C8—H8A	0.9300	O2W—H2WA	0.837 (19)
N4—H4B	0.895 (10)	O2W—H2WB	0.87 (4)
N4—H4C	0.90 (3)	O1W—H1WA	0.84 (6)
N3—C7	1.374 (4)	O1W—H1WB	0.849 (18)
			
C9—N1—C1	117.4 (2)	C7—N3—H3C	120 (3)
C10—N2—C6	117.2 (2)	H3B—N3—H3C	114 (4)
N2—C10—C11	120.1 (2)	C4—C5—C6	120.3 (3)
N2—C10—C9	121.2 (2)	C4—C5—H5A	119.9
C11—C10—C9	118.7 (2)	C6—C5—H5A	119.9
C12—C11—C10	121.5 (3)	N2—C6—C1	121.9 (2)
C12—C11—H11A	119.3	N2—C6—C5	119.2 (3)
C10—C11—H11A	119.3	C1—C6—C5	118.8 (2)
N1—C1—C6	121.2 (2)	C3—C2—C1	120.1 (3)
N1—C1—C2	119.7 (3)	C3—C2—H2A	119.9
C6—C1—C2	119.1 (3)	C1—C2—H2A	119.9
N4—C12—C11	121.7 (3)	C8—C7—N3	121.5 (3)
N4—C12—C7	118.4 (3)	C8—C7—C12	119.5 (3)
C11—C12—C7	119.8 (3)	N3—C7—C12	118.9 (3)
C7—C8—C9	122.2 (3)	C5—C4—C3	120.9 (3)
C7—C8—H8A	118.9	C5—C4—H4A	119.6
C9—C8—H8A	118.9	C3—C4—H4A	119.6
N1—C9—C8	120.5 (3)	C2—C3—C4	120.8 (3)
N1—C9—C10	121.1 (2)	C2—C3—H3A	119.6
C8—C9—C10	118.4 (2)	C4—C3—H3A	119.6
C12—N4—H4B	113 (3)	H4WB—O4W—H4WA	108 (2)
C12—N4—H4C	118 (2)	H3WB—O3W—H3WA	105 (2)
H4B—N4—H4C	128 (4)	H2WA—O2W—H2WB	109 (2)
C7—N3—H3B	117 (3)	H1WA—O1W—H1WB	112 (3)
			
C6—N2—C10—C11	179.8 (2)	N1—C1—C6—N2	0.5 (4)
C6—N2—C10—C9	0.4 (4)	C2—C1—C6—N2	179.4 (3)
N2—C10—C11—C12	−179.2 (2)	N1—C1—C6—C5	−179.0 (3)
C9—C10—C11—C12	0.2 (4)	C2—C1—C6—C5	0.0 (4)
C9—N1—C1—C6	−0.3 (4)	C4—C5—C6—N2	−179.2 (3)
C9—N1—C1—C2	−179.2 (2)	C4—C5—C6—C1	0.2 (4)
C10—C11—C12—N4	−176.0 (3)	N1—C1—C2—C3	179.0 (3)
C10—C11—C12—C7	0.2 (4)	C6—C1—C2—C3	0.0 (4)
C1—N1—C9—C8	−179.3 (2)	C9—C8—C7—N3	175.9 (3)
C1—N1—C9—C10	0.2 (4)	C9—C8—C7—C12	0.6 (4)
C7—C8—C9—N1	179.2 (2)	N4—C12—C7—C8	175.8 (3)
C7—C8—C9—C10	−0.2 (4)	C11—C12—C7—C8	−0.6 (4)
N2—C10—C9—N1	−0.3 (3)	N4—C12—C7—N3	0.4 (4)
C11—C10—C9—N1	−179.6 (3)	C11—C12—C7—N3	−176.0 (3)
N2—C10—C9—C8	179.2 (3)	C6—C5—C4—C3	−0.5 (4)
C11—C10—C9—C8	−0.2 (3)	C1—C2—C3—C4	−0.3 (5)
C10—N2—C6—C1	−0.5 (4)	C5—C4—C3—C2	0.5 (5)
C10—N2—C6—C5	179.0 (2)		

### Hydrogen-bond geometry (Å, °)

**Table:**

*D*—H···*A*	*D*—H	H···*A*	*D*···*A*	*D*—H···*A*
N4—H4B···O2W^i^	0.895 (10)	2.218 (12)	3.105 (5)	171 (3)
N4—H4C···N4^ii^	0.90 (3)	2.58 (3)	3.198 (3)	126 (3)
N3—H3B···O1W^iii^	0.906 (10)	2.165 (16)	3.048 (6)	165 (4)
N3—H3C···N3^ii^	0.895 (11)	2.33 (2)	3.122 (4)	147 (3)
O4W—H4WA···O4W^iv^	0.855 (19)	2.017 (19)	2.871 (3)	176 (4)
O4W—H4WB···N2	0.867 (17)	1.924 (19)	2.787 (3)	173 (4)
O3W—H3WB···N1	0.872 (19)	1.96 (3)	2.801 (3)	161 (6)
O2W—H2WA···O4W^v^	0.84 (2)	2.01 (2)	2.843 (4)	178 (5)
O1W—H1WA···O1W^vi^	0.84 (6)	2.15 (6)	2.860 (7)	142 (6)
O2W—H2WB···O2W^vii^	0.87 (4)	1.97 (4)	2.812 (5)	161 (3)
O1W—H1WB···O3W	0.85 (3)	2.09 (3)	2.882 (6)	155 (5)

Symmetry codes: (i) *x*, *y*, *z*−1; (ii) −*x*+1/2, *y*, *z*−1/2; (iii) −*x*+1, −*y*+1, *z*−1/2; (iv) −*x*+1, −*y*, *z*−1/2; (v) −*x*+1, −*y*, *z*+1/2; (vi) −*x*+3/2, *y*, *z*+1/2; (vii) −*x*+1/2, *y*, *z*+1/2.

## References

[bb1] Brownstein, S. K. & Enright, G. D. (1995). *Acta Cryst.* C**51**, 1579–1581.

[bb2] Bruker (2001). *SAINT* and *SMART* Bruker AXS Inc., Madison, Wisconsin, USA.

[bb4] Chłopek, K., Bill, E., Weyhermüller, T. & Wieghardt, K. (2005). *Inorg. Chem.* **44**, 7087–7098. 10.1021/ic050829k 16180871

[bb3] Doyle, R. P., Kruger, P. E., Mackie, P. R. & Nieuwenhuyzen, M. (2001). *Acta Cryst.* C**57**, 104–105. 10.1107/s0108270100014712 11173415

[bb5] Sheldrick, G. M. (1996). *SADABS* University of Göttingen, Germany.

[bb6] Sheldrick, G. M. (2008). *Acta Cryst.* A**64**, 112–122. 10.1107/S0108767307043930 18156677

